# Supplementary material for: Dysregulation of Redox Status in Urinary Bladder Cancer Patients
Source: Cancers (Basel). 2020 May 21;12(5):1296. doi: 10.3390/cancers12051296 (PMC7280975; doi:10.3390/cancers12051296)
Supplement: Supplementary file 1 [file cancers-12-01296-s001.zip › cancers-794156-suppl-final/cancers-794156-suppl-final.docx]

Article

Dysregulation of Redox Status in Urinary Bladder Cancer Patients

Edyta Reszka, Monika Lesicka, Edyta Wieczorek, Ewa Jabłońska, Beata Janasik, Maciej Stępnik, Tomasz Konecki and Zbigniew Jabłonowski

Supplementary Materials:

**Table S2.** Cytoprotective genes expression and DNA damage in separated groups of the Urinary bladder cancer patients and the Controls according to gender.

| **Variable** | **Urinary bladder cancer** | | | **Control** | | |
| --- | --- | --- | --- | --- | --- | --- |
|  | **Women**  ***N* = 36** | **Men**  ***N* = 86** | ***p*-Value** | **Women**  ***N* = 34** | **Men**  ***N* = 81** | ***p*-Value** |
| Age | 62.944 | 62.849 | 0.753 | 66.147 | 66.475 | 0.705 |
| BMI | 26.322 | 27.699 | 0.132^1^ | 24.922 | 27.572 | **0.000^1^** |
| Se^2^ | 70.439 | 66.293 | 0.205^1^ | 77.412 | 73.506 | 0.195 |
| *NRF2* | 8.581 | 8.524 | 0.395^1^ | 8.445 | 8.449 | 0.856^1^ |
| *KEAP1* | 5.533 | 5.438 | 0.156^1^ | 6.039 | 5.768 | **0.001^1^** |
| *MAFG* | 7.991 | 7.961 | 0.598^1^ | 8.013 | 7.970 | 0.079 |
| *ABCC4* | 5.960 | 5.920 | 0.666 | 5.975 | 5.972 | 0.852 |
| *GCLC* | 6.463 | 6.411 | 0.550 | 6.254 | 6.330 | 0.245^1^ |
| *GCLM* | 7.843 | 7.830 | 0.212 | 7.898 | 7.886 | 0.443 |
| *GSR* | 7.211 | 7.180 | 0.980 | 7.219 | 7.306 | **0.001** |
| *GSTP1* | 10.427 | 10.380 | 0.365^1^ | 10.470 | 10.400 | 0.079^1^ |
| *HMOX1* | 8.606 | 8.667 | 0.886 | 8.845 | 8.809 | 0.493^1^ |
| *MMP9* | 8.316 | 8.542 | 0.167^1^ | 8.101 | 8.242 | 0.489 |
| *NQO1* | 5.460 | 5.461 | 0.990^1^ | 5.300 | 5.754 | **0.000^1^** |
| *OGG1* | 3.249 | 3.100 | 0.119^1^ | 3.803 | 3.431 | **0.000** |
| *PRDX1* | 9.522 | 9.478 | 0.337^1^ | 9.620 | 9.498 | **0.000^1^** |
| *SOD1* | 9.078 | 9.073 | 0.775 | 9.064 | 9.064 | 0.604 |
| *SOD2* | 10.989 | 11.005 | 0.902 | 11.016 | 10.872 | 0.083 |
| *SRXN1* | 7.922 | 7.953 | 0.751^1^ | 7.916 | 7.857 | 0.315 |
| *UGT1A6* | 3.365 | 3.405 | 0.823^1^ | 3.852 | 3.451 | **0.013** |
| *GPX1* | 11.827 | 11.817 | 0.569 | 11.744 | 11.715 | 0.695 |
| *SELT* | 7.416 | 7.353 | 0.197^1^ | 7.417 | 7.341 | **0.029** |
| *SEP15* | 9.160 | 9.087 | **0.022^1^** | 9.151 | 9.063 | **0.001** |
| *SELW1* | 6.158 | 6.017 | **0.052** | 6.675 | 6.398 | **0.000** |
| *TRXR1* | 5.184 | 5.137 | 0.624^1^ | 5.049 | 5.222 | **0.013^1^** |
| *DNMT1* | 6.787 | 6.658 | **0.039^1^** | 6.836 | 6.882 | 0.390^1^ |
| *DNMT3A* | 6.406 | 6.279 | 0.210 | 6.308 | 6.450 | **0.022^1^** |
| *SIRT1* | 6.233 | 6.174 | 0.275^1^ | 6.340 | 6.229 | **0.038** |
| Tail DNA^3^ (%) | 6.693 | 6.990 | 0.645 | 4.409 | 4.814 | 0.600 |
| Ox Tail DNA^4^ (%) | 11.094 | 10.689 | 0.544 | 7.031 | 7.263 | 0.720 |

The *p*-values were calculated using the Student’s t-test or u Man Whitney test; ^1^variables were normally distributed ^2^plasma selenium level (µg/L); the percentage of DNA damage^3^ and oxidative DNA damage^4^ in tail; The significant *p*-values are marked in bold.

**Table S3.** Cytoprotective genes expression and DNA damage in separated groups of the Urinary bladder cancer patients and the Controls according to smoking habit.

| **Variable** | **Urinary Bladder Cancer** | | | **Control** | | |
| --- | --- | --- | --- | --- | --- | --- |
|  | **No smokers**  **N=80** | **Smokers**  **N=42** | ***p-*Value** | **No smokers**  **N=100** | **Smokers**  **N=15** | ***p*-Value** |
| Age | 63.513 | 61.667 | 0.252 | 66.515 | 65.467 | 0.330 |
| BMI | 27.893 | 26.149 | **0.046^1^** | 26.676 | 27.537 | 0.341^1^ |
| Se^2^ | 68.974 | 64.740 | 0.177**^1^** | 74.771 | 73.927 | 0.937 |
| *NRF2* | 8.546 | 8.531 | 0.820**^1^** | 8.445 | 8.463 | 0.954^1^ |
| *KEAP1* | 5.485 | 5.430 | 0.385**^1^** | 5.862 | 5.753 | 0.341^1^ |
| *MAFG* | 7.978 | 7.954 | 0.659**^1^** | 7.987 | 7.955 | 0.787 |
| *ABCC4* | 5.964 | 5.871 | 0.191 | 5.979 | 5.930 | 0.430 |
| *GCLC* | 6.457 | 6.368 | 0.141 | 6.324 | 6.195 | 0.142^1^ |
| *GCLM* | 7.845 | 7.812 | 0.319 | 7.889 | 7.892 | 0.904 |
| *GSR* | 7.203 | 7.162 | 0.527 | 7.270 | 7.348 | 0.778 |
| *GSTP1* | 10.411 | 10.361 | 0.321^1^ | 10.434 | 10.331 | 0.057^1^ |
| *HMOX1* | 8.704 | 8.545 | 0.005 | 8.835 | 8.716 | 0.094^1^ |
| *MMP9* | 8.457 | 8.510 | 0.738^1^ | 8.154 | 8.516 | 0.069 |
| *NQO1* | 5.471 | 5.442 | 0.772**^1^** | 5.626 | 5.577 | 0.718^1^ |
| *OGG1* | 3.185 | 3.068 | 0.202**^1^** | 3.568 | 3.360 | **0.050** |
| *PRDX1* | 9.508 | 9.459 | 0.252^1^ | 9.545 | 9.465 | 0.067^1^ |
| *SOD1* | 9.096 | 9.034 | 0.189 | 9.085 | 8.925 | **0.016** |
| *SOD2* | 11.027 | 10.948 | 0.550 | 10.906 | 10.969 | 0.721 |
| *SRXN1* | 7.986 | 7.864 | 0.195**^1^** | 7.856 | 7.995 | 0.542 |
| *UGT1A6* | 3.414 | 3.353 | 0.717**^1^** | 3.657 | 2.967 | **0.007** |
| *GPX1* | 11.903 | 11.661 | **0.011** | 11.754 | 11.521 | **0.030** |
| *SELT* | 7.386 | 7.343 | 0.368**^1^** | 7.377 | 7.274 | 0.346 |
| *SEP15* | 9.134 | 9.061 | 0.017^1^ | 9.098 | 9.028 | 0.071 |
| *SELW1* | 6.055 | 6.064 | 0.893 | 6.531 | 6.139 | **0.028** |
| *TRXR1* | 5.109 | 5.230 | 0.187**^1^** | 5.158 | 5.255 | 0.311^1^ |
| *DNMT1* | 6.672 | 6.743 | 0.239**^1^** | 6.885 | 6.761 | 0.084^1^ |
| *DNMT3A* | 6.280 | 6.386 | 0.227 | 6.394 | 6.503 | 0.198^1^ |
| *SIRT1* | 6.194 | 6.187 | 0.907**^1^** | 6.270 | 6.206 | 0.375 |
| Tail DNA^3^ (%) | 6.740 | 7.212 | 0.303 | 4.751 | 4.316 | 0.205 |
| Ox Tail DNA^4^ (%) | 10.584 | 11.235 | 0.261 | 7.203 | 7.141 | 0.950 |

The *p*-values were calculated using the Student’s *t*-test or U Man Whitney test; ^1^variables were normally distributed; The significant p-values are marked in bold; ^2^plasma selenium level (µg/L); the percentage of DNA damage^3^ and oxidative DNA damage^4^ in tail.

**Table S4.** Correlation between expression of NRF2 and selected cytoprotective NRF2-related genes.

| **Gene** | ***NRF2***  **r-Coefficient^1^** | |
| --- | --- | --- |
|  | **Urinary Bladder Cancer** | **Control** |
| *KEAP1* | **0.353** | -0.027 |
| *MAFG* | **0.647** | **0.518** |
| *ABCC4* | **0.320** | **0.283** |
| *GCLC* | **0.317** | **0.277** |
| *GCLM* | **0.279** | **0.330** |
| *GSR* | **0.564** | **0.488** |
| *GSTP1* | **0.358** | **0.239** |
| *HMOX1* | **0.535** | **0.401** |
| *MMP9* | **0.626** | **0.484** |
| *NQO1* | 0.062 | 0.089 |
| *OGG1* | 0.143 | 0.036 |
| *PRDX1* | **0.233** | **0.191** |
| *SOD1* | **0.193** | **0.248** |
| *SOD2* | **0.693** | **0.571** |
| *SRXN1* | **0.548** | **0.395** |
| *UGT1A6* | 0.121 | -0.017 |
| *GPX1* | **0.371** | **0.299** |
| *SELT* | **0.214** | **0.349** |
| *SEP15* | **0.350** | **0.372** |
| *SEPW1* | -0.120 | -0.065 |
| *TRXR1* | **0.512** | **0.395** |
| *DNMT1* | 0.093 | -0.029 |
| *DNMT3A* | **0.519** | **0.538** |
| *SIRT1* | 0.139 | 0.167 |

^1^r-coefficient - Spearman’s rank correlation coefficient; Values in bold are significant *p* < 0.05.

**Table S5.** Association between cytoprotective genes expression and DNA damage.

| **Gene** | **Urinary bladder cancer** | | | **Control** | | |
| --- | --- | --- | --- | --- | --- | --- |
|  | **Beta-Coefficient(ß)** | ***p*-value^1^** | ***p*-value^2^** | **Beta-Coefficient(ß)** | ***p*-Value^1^** | ***p*-Value^2^** |
| *NRF2* | 0.204 | **0.025** | 0.242 | -0.098 | 0.301 | 0.536 |
| *KEAP1* | -0.203 | **0.025** | 0.771 | 0.201 | **0.029** | **0.001** |
| *MAFG* | 0.226 | **0.013** | 0.384 | -0.258 | **0.006** | 0.803 |
| *ABCC4* | 0.038 | 0.674 | 0.932 | -0.029 | 0.760 | 0.881 |
| *GCLC* | 0.069 | 0.443 | **0.005** | 0.154 | **0.096** | 0.350 |
| *GCLM* | -0.240 | **0.010** | **0.001** | -0.002 | 0.982 | 0.720 |
| *GSR* | 0.174 | **0.060** | 0.764 | 0.005 | 0.955 | 0.177 |
| *GSTP1* | 0.056 | 0.548 | 0.507 | 0.018 | 0.789 | **0.041** |
| *HMOX1* | 0.005 | 0.955 | 0.720 | 0.037 | 0.692 | **0.039** |
| *MMP9* | 0.150 | 0.105 | 0.650 | -0.141 | 0.140 | 0.410 |
| *NQO1* | 0.181 | **0.053** | 0.216 | -0.041 | 0.636 | 0.937 |
| *OGG1* | -0.080 | 0.386 | 0.495 | -0.083 | 0.369 | **0.005** |
| *PRDX1* | 0.243 | **0.006** | 0.063 | 0.121 | 0.164 | 0.512 |
| *SOD1* | 0.268 | **0.002** | **0.038** | 0.136 | 0.132 | **0.037** |
| *SOD2* | 0.081 | 0.376 | 0.794 | -0.091 | 0.338 | 0.933 |
| *SRXN1* | 0.049 | 0.596 | 0.810 | -0.095 | 0.323 | 0.377 |
| *UGT1A6* | 0.153 | 0.109 | 0.332 | -0.070 | 0.459 | 0.465 |
| *GPX1* | 0.041 | 0.641 | 0.425 | -0.105 | 0.270 | 0.316 |
| *SELT* | 0.030 | 0.735 | **0.014** | 0.046 | 0.619 | 0.502 |
| *SEP15* | 0.010 | 0.910 | 0.112 | 0.022 | 0.803 | 0.507 |
| *SEPW1* | 0.146 | 0.112 | 0.913 | 0.047 | 0.596 | **0.017** |
| *TRXR1* | -0.004 | 0.963 | 0.432 | -0.055 | 0.557 | 0.236 |
| *DNMT1* | 0.016 | 0.862 | 0.783 | 0.123 | 0.192 | 0.357 |
| *DNMT3A* | -0.040 | 0.661 | 0.559 | -0.127 | 0.180 | 0.078 |
| *SIRT1* | -0.022 | 0.806 | 0.391 | 0.165 | 0.079 | 0.620 |

^1^ The P-values calculated by linear regression adjusted by age, gender, BMI. alcohol and smoking habits, separately for each gene; ^2^ The p-values calculated by one- way ANOVA according to tertiles of Tail DNA (%); Values in bold are significant *p* < 0.05.

**Table S6.** Association between cytoprotective genes expression and oxidative DNA damage.

| **Gene** | **Urinary bladder cancer** | | | **Control** | | |
| --- | --- | --- | --- | --- | --- | --- |
|  | **Beta-Coefficient(ß)** | **p-value^1^** | **p-value^2^** | **Beta-Coefficient(ß)** | **p-Value^1^** | **p-Value^2^** |
| *NRF2* | 0.073 | 0.430 | 0.280 | -0.055 | 0.559 | 0.726 |
| *KEAP1* | -0.164 | 0.071 | 0.115 | 0.156 | 0.088 | 0.073 |
| *MAFG* | 0.080 | 0.389 | 0.521 | -0.194 | **0.037** | **0.029** |
| *ABCC4* | 0.038 | 0.680 | 0.459 | -0.058 | 0.539 | 0.703 |
| *GCLC* | 0.027 | 0.763 | 0.601 | 0.130 | 0.157 | 0.257 |
| *GCLM* | -0.212 | **0.023** | 0.668 | -0.028 | 0.762 | 0.661 |
| *GSR* | 0.049 | 0.597 | 0.514 | -0.065 | 0.489 | 0.511 |
| *GSTP1* | 0.050 | 0.589 | 0.494 | -0.059 | 0.511 | 0.618 |
| *HMOX1* | -0.030 | 0.737 | 0.566 | -0.006 | 0.948 | 0.876 |
| *MMP9* | 0.035 | 0.704 | 0.787 | -0.165 | 0.079 | 0.216 |
| *NQO1* | 0.064 | 0.499 | 0.113 | 0.029 | 0.738 | 0.935 |
| *OGG1* | 0.019 | 0.837 | 0.860 | -0.068 | 0.456 | 0.952 |
| *PRDX1* | 0.185 | **0.039** | 0.753 | 0.057 | 0.507 | 0.610 |
| *SOD1* | 0.102 | 0.256 | 0.806 | 0.140 | 0.118 | **0.015** |
| *SOD2* | -0.008 | 0.933 | 0.622 | -0.077 | 0.415 | 0.709 |
| *SRXN1* | 0.025 | 0.784 | 0.624 | -0.063 | 0.506 | 0.633 |
| *UGT1A6* | 0.088 | 0.359 | 0.101 | -0.068 | 0.467 | 0.896 |
| *GPX1* | 0.065 | 0.469 | 0.682 | -0.077 | 0.411 | 0.777 |
| *SELT* | -0.098 | 0.271 | 0.614 | 0.106 | 0.248 | 0.831 |
| *SEP15* | -0.013 | 0.885 | 0.732 | 0.064 | 0.469 | 0.777 |
| *SEPW1* | 0.034 | 0.717 | 0.306 | 0.030 | 0.729 | 0.677 |
| *TRXR1* | -0.119 | 0.193 | 0.419 | -0.054 | 0.562 | 0.883 |
| *DNMT1* | -0.075 | 0.406 | 0.890 | 0.090 | 0.338 | 0.067 |
| *DNMT3A* | -0.151 | 0.100 | 0.253 | -0.140 | 0.134 | 0.454 |
| *SIRT1* | -0.032 | 0.724 | 0.398 | 0.174 | 0.060 | **0.043** |

^1^ The *p*-values calculated by linear regression adjusted by age, gender, BMI, alcohol and smoking habits, separately for each gene; ^2^ P values calculated by one- way ANOVA according to tertiles of Oxidative Tail DNA (%); Values in bold are significant *p* < 0.05.

**Table S7.** Categories of distribution of gene expression and DNA damage.

| Redox parameter | Group | **Min.** | **Max.** | **Low**  **Tertile 1** | ***n*** | **Medium**  **Tertile 2** | ***n*** | **High**  **Tertile 3** | ***n*** |
| --- | --- | --- | --- | --- | --- | --- | --- | --- | --- |
|  |  |  |  | **≤33rd^1^** |  | **33rd-66th^1^** |  | **> 66th th^1^** |  |
| *PRDX1* | UBC | 8.85 | 10.34 | 9.47 | 66 | 9.47-9.61 | 16 | 9.61 | 40 |
|  | Co | 9.08 | 9.88 | 9.47 | 39 | 9.47-9.61 | 37 | 9.61 | 39 |
| *SRXN1* | UBC | 6.32 | 9.09 | 7.68 | 35 | 7.68-8.03 | 35 | 8.03 | 52 |
|  | Co | 5.31 | 9.40 | 7.68 | 38 | 7.68-8.03 | 39 | 8.03 | 38 |
| Tail DNA (%)^2^ | UBC | 1.64 | 14.24 | 3.74 | 7 | 3.74-5.1 | 24 | 5.1 | 91 |
|  | Co | 2.09 | 10.13 | 3.74 | 8 | 3.74-5.1 | 6 | 5.1 | 108 |
| Ox Tail DNA (%)^3^ | UBC | 4.25 | 21.58 | 6.21 | 40 | 6.21-7.37 | 37 | 7.37 | 38 |
|  | Co | 3.44 | 13.57 | 6.21 | 39 | 6.21-7.37 | 38 | 7.37 | 38 |

^1^The DNA damages and gene expression level were classified as “low”, “medium” or “high” according to the genes expression level, % Tail DNA damages, Oxidative %Tail DNA damages in control group. The “low” correspond to the values ≤33rd percentile, “medium” between the 33rd percentile and the 66th percentile and “high” for the values >66th percentile; UBC - Urinary bladder cancer; Co - Control group; the percentage of DNA damage^2^ and oxidative DNA damage^3^ in tail.

| 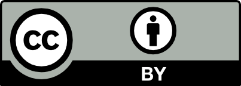 | © 2020 by the authors. Submitted for possible open access publication under the terms and conditions of the Creative Commons Attribution (CC BY) license (http://creativecommons.org/licenses/by/4.0/). |
| --- | --- |
